# Network analysis of quantitative proteomics on asthmatic bronchi: effects of inhaled glucocorticoid treatment

**DOI:** 10.1186/1465-9921-12-124

**Published:** 2011-09-22

**Authors:** Serena E O'Neil, Brigita Sitkauskiene, Agne Babusyte, Algirda Krisiukeniene, Kristina Stravinskaite-Bieksiene, Raimundas Sakalauskas, Carina Sihlbom, Linda Ekerljung, Elisabet Carlsohn, Jan Lötvall

**Affiliations:** 1 Krefting Research Centre, Department of Internal Medicine, University of Gothenburg, Sweden; 2 Department of Pulmonology and Immunology, Lithuanian University of Health Sciences, Lithuania; 3 Proteomics Core Facility, Sahlgrenska Academy, University of Gothenburg, Sweden

**Keywords:** asthma, quantitative proteomics, bronchial biopsies, glucocorticoid, network analysis, iTRAQ, isobaric tags for relative and absolute quantitation, Ingenuity Pathway Analysis

## Abstract

**Background:**

Proteomic studies of respiratory disorders have the potential to identify protein biomarkers for diagnosis and disease monitoring. Utilisation of sensitive quantitative proteomic methods creates opportunities to determine individual patient proteomes. The aim of the current study was to determine if quantitative proteomics of bronchial biopsies from asthmatics can distinguish relevant biological functions and whether inhaled glucocorticoid treatment affects these functions.

**Methods:**

Endobronchial biopsies were taken from untreated asthmatic patients (*n *= 12) and healthy controls (*n *= 3). Asthmatic patients were randomised to double blind treatment with either placebo or budesonide (800 μg daily for 3 months) and new biopsies were obtained. Proteins extracted from the biopsies were digested and analysed using isobaric tags for relative and absolute quantitation combined with a nanoLC-LTQ Orbitrap mass spectrometer. Spectra obtained were used to identify and quantify proteins. Pathways analysis was performed using Ingenuity Pathway Analysis to identify significant biological pathways in asthma and determine how the expression of these pathways was changed by treatment.

**Results:**

More than 1800 proteins were identified and quantified in the bronchial biopsies of subjects. The pathway analysis revealed acute phase response signalling, cell-to-cell signalling and tissue development associations with proteins expressed in asthmatics compared to controls. The functions and pathways associated with placebo and budesonide treatment showed distinct differences, including the decreased association with acute phase proteins as a result of budesonide treatment compared to placebo.

**Conclusions:**

Proteomic analysis of bronchial biopsy material can be used to identify and quantify proteins using highly sensitive technologies, without the need for pooling of samples from several patients. Distinct pathophysiological features of asthma can be identified using this approach and the expression of these features is changed by inhaled glucocorticoid treatment. Quantitative proteomics may be applied to identify mechanisms of disease that may assist in the accurate and timely diagnosis of asthma.

**Trial registration:**

ClinicalTrials.gov registration NCT01378039

## Background

Asthma is one of the most common chronic diseases in the world and poses a vast burden on society with limited new treatments being developed [1,2]. Understanding asthma has proven to be a challenge, although immunological tools and genomic studies have started to dissect mechanisms of asthma using animal models and cell cultures expressing certain limited characteristics of the disease. However, human asthma is not totally reflected in these systems, therefore the identification of the mechanisms of human disease and their interactions is needed.

In humans, the investigation of asthma mechanisms is commonly explored using clinical examinations and relatively small patient samples. The gene and protein investigation of asthma pathogenesis has progressed from local to global studies. Genomic efforts to identify genes and mechanisms related to asthma pathogenesis in humans have identified multiple areas of interest, as reviewed by Rolph *et al *[3]. While genomic studies provide a wealth of information, it is their translated products, the proteins, which direct cellular functions. The global proteome in asthma has yet to be thoroughly investigated, which may more efficiently identify mechanisms and markers of asthma.

To date, the proteomic studies of asthma fall into three broad categories; tissues, fluids and cells, each posing different analytical challenges. The majority of studies concerning asthma have been conducted on bronchoalveolar lavage fluid (BALF) and lung tissue of mouse models [4-7], which can only represent certain features of asthma. The few efforts in human asthma have mainly centred around cell cultures [8], sputum [9], BALF [10], T lymphocytes [11] and more recently, exhaled breath condensate [12]. While these samples present aspects of asthma, they do not totally reflect the site of disease, the bronchi. These proteomics studies have generated a list of identified proteins using methods like SDS-PAGE, 2-DE, LC-MS/MS, SELDI-TOF and MALDI-TOF [13]. Despite being successively used in other fields [14-18], the use of quantitative proteomics, specifically isobaric tags for relative and absolute quantitation (iTRAQ^®^) technology, has not been applied to understanding the complexity of asthma.

In the present study, we aimed to analyse the global proteome of bronchial biopsies taken from asthmatics and compare it to the proteome of healthy controls. In addition, the effects of budesonide treatment on the asthmatic bronchial proteome were also examined. Utilising sensitive, high throughput iTRAQ^® ^technology to quantify proteins, the resulting proteome was analysed using Ingenuity Pathways Analysis (IPA). Distinct proteome differences were observed in asthmatics compared to controls, with increased acute phase response and actin based signalling. The effects of glucocorticoid treatment were seen with changes in the functions displayed between placebo treated and budesonide treated patients, specifically, the reduction of the acute phase association and increased cellular processes. This study is the first to present quantitative proteomics of small clinically relevant tissue from asthmatic individuals, as well as determining the effects of inhaled glucocorticoid treatment on this proteome.

## Methods

### Objectives

1. Determine if quantitative proteomic analysis of individual biopsies can identify biologically relevant mechanisms of disease.

2. Determine if the proteome of bronchial biopsies are modified with inhaled glucocorticoid treatment.

### Participant Selection and Demographics

The proteomics study was conducted on 12 non-smoking out-patients, aged 40-80 years, with a clinical history of asthma. Patients had stable, mild to moderate asthma, in accordance with the Global Initiative for Asthma guidelines [19]. Three non-smoking, non-asthmatic subjects were used as controls. Subjects were recruited and diagnosed by a pulmonologist based on clinical history, lung function measurements and methacholine provocation prior to inclusion in the study.

To be included in the study, subjects were required to have abstained from inhaled and oral glucocorticoids six weeks prior to the study and long acting bronchodilators four weeks prior. Pregnancy was a criterion for exclusion. Control subjects were required to have a baseline FEV_1 _> 80% of predicted. Subjects without hypoxemia were included.

No subject had exacerbations or respiratory infections in the four weeks prior to their first visit or any respiratory disorders other than asthma. Subjects who had serious uncontrolled diseases, had any evidence of an interfering condition by X-ray or had participated in another clinical study in the two months prior, were excluded from participating in the current study. Subjects receiving treatment with cromolyn sodium or nedocromyl, oral beta2 - agonists, long acting anti-cholinergic or leukotriene modifiers were also excluded.

### Ethics Statement

The study was approved by the Regional Bioethics Committee of Kaunas University of Medicine (protocol no. 48/2004) with written informed consent received from all participants. The study is registered with ClinicalTrials.gov (Identifier: NCT01378039).

### Study Design

The study was performed in a double blind, placebo controlled design. The patients with asthma were randomised to three months treatment with either inhaled budesonide (400 μg twice daily) or placebo twice daily. Salbutamol was allowed as needed.

Spirometry was performed and methacholine responsiveness measured using a CustovitM pneumotachometric spirometer (Custo Med, Germany). Fractional exhaled nitric oxide (F_E_NO) was measured using the Niox Mino^® ^analyzer (Aerocrine AB, Sweden) at a flow rate of 50 mL/s. An average of three measurements was used for analysis.

Skin prick tests (SPT) were performed using standard allergens, *Dermatophagoides pteronyssinus, Dermatophagoides farinae*, cat epithelia, mixed mould, mixed grasses, Betulaceae and mixed weeds (Stallergenes, France).

### Bronchial Biopsy

A bronchoscope (Olympus BF-B3R) and forceps (Olympus FB-20C -1, 2.6 channel diameter, length 1050 mm; both from Olympus Corporation, Tokyo, Japan) were used to take endobronchial biopsies (1-2 mm) from the left upper, middle and lower lobes, segmental and subsegmental bronchus. Biopsies were immediately transferred to individual microtubes and snap frozen with liquid nitrogen, before being stored at -80°C. Control subjects (healthy non-smokers) had bronchial biopsies taken on one occasion only. When consenting, patients had biopsies taken prior to, and following, the treatment period.

### Summary of Proteomics Workflow

**Figure 1:**
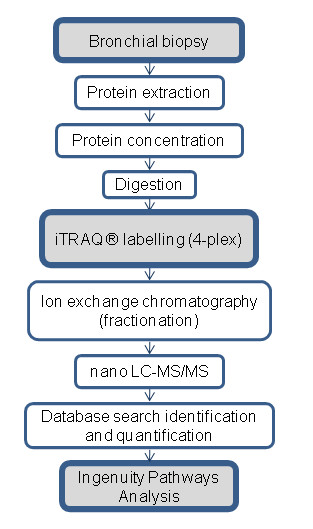
**Schematic overview of the experimental workflow**. The workflow used in the proteomics analysis of human endobronchial biopsies. Proteins were extracted and digested, before the peptides were labelled with iTRAQ**^® ^**Reagents in a four-plex set. Each four-plex, containing one reference pool and three samples, were fractionated using ion exchange chromatography. Each fraction was then subjected to nano LC-MS/MS. The resulting spectra were searched for identification and quantification. The identified and quantified proteins were then analysed using Ingenuity Pathways Analysis.

An overview of the workflow is presented in Figure 1. Briefly, protein extracted from bronchial biopsies was digested into peptides, which were labelled with iTRAQ^® ^Reagents. A group of four labelled samples; three individual samples and one reference pool, were mixed to form a four-plex set. The complexity of each four-plex set was reduced by fractionation using strong cation exchange chromatography (SCX). Each fraction was then subjected to nano LC-MS/MS. The resulting spectra were searched in a database for protein identities and the reporter ions from the iTRAQ^® ^Reagents were used to quantify proteins.

### Protein Extraction

One to two middle lobe biopsies from each subject were pooled and used for the proteomic study. Pooling of samples into larger disease groups was not performed in order to retain individual proteome information. Preliminary proteomics determined that the amount of protein extracted from individual biopsies was suitable for iTRAQ^® ^analysis. Blinded endobronchial biopsies were washed by adding 100 μl chilled milliQ water and vortexing. The biopsies were spun and the water discarded. Fifty microlitres of lysis buffer (8 M urea, 4% CHAPS, 0.1% SDS, 10 mM EDTA, 5 mM TCEP) was added to the biopsies before being sonicated in a water bath for 2 × 20 secs with a 20 sec rest between bursts. A pellet pestle (Sigma-Aldrich Sweden AB, Sweden) was used to homogenise the biopsies, with 50 μl of lysis buffer used to rinse the pestle. The homogenate was incubated for 1 hr at room temperature with shaking, followed by centrifugation at 16 000 × g for 10 min. The tube was then stored at -20°C prior to tryptic digestion. The tube was centrifuged briefly prior to extraction of the supernatant for further applications. The protein concentration of each sample was determined using the Pierce BCA Protein Assay (Thermo Fisher Scientific, IL, USA). A reference pool was made by pooling aliquots from remaining extracted protein samples. This common sample allows for the normalisation and comparison of all sets.

### iTRAQ Labelling of Peptides

65 μg of protein from each sample was withdrawn and diluted four times with dissolution solution (0.5 M TEAB (triethylammonium bicarbonate) and milli-Q water)) and the samples were reduced with TCEP ((tris(2-carboxyethyl)phosphine) and alkylated with MMTS (methyl methanethiolsulfonate) before digestion with trypsin (Promega Corporation, WI, USA). Each sample in the four-plex set, consisting of one pooled reference sample and three individual patient samples, was then labelled with the iTRAQ^® ^Reagents 114, 115, 116, and 117 respectively, following manufacturer's instructions (Applied Biosystems, Foster City, CA, USA). The reference pool was always labelled with iTRAQ^® ^Reagent 114, while the patient samples were randomised to the remaining three iTRAQ^® ^Reagents within the experiment.

### Strong Cation Exchange (SCX) Fractionation of iTRAQ-labelled Peptides

SCX chromatography was used to remove unbound iTRAQ^® ^Reagents and to reduce the sample complexity by fractionation. The concentrated peptides were acidified by 10% formic acid and diluted with SCX solvent A (25 mM ammonium formate, pH 2.8, 20% ACN (acetonitrile)) and injected onto a PolySULFOETHYL A SCX column (PolyLC Incorporated, MD, USA) (2.1 mm i.d. × 10 cm length, 5 μm particle size, 300 Å pore size). SCX chromatography and fractionation was carried out on an ÄKTA purifier system (GE Healthcare, Buckinghamshire, UK) at 0.25 mL/min flow rate using the following gradient: 0% B (500 mM ammonium formate, pH 2.8, 20% ACN) for 5 min; 0-40% B for 20 min; 40-100% B for 10 min and 100% B held for 10 min. UV absorbance at 254 and 280 nm was monitored, while fractions were collected at 0.5 mL intervals and dried down in a SpeedVac. The 15 peptide-containing fractions were desalted on PepClean C-18 Spin Columns according to manufacturer's instructions (Thermo Fisher Scientific Inc, IL, USA).

### LC-MS/MS Analysis on LTQ-Orbitrap

The desalted and dried fractions were reconstituted into 0.1% formic acid and analysed on an LTQ-Orbitrap XL (Thermo Fisher Scientific) interfaced with an in-house constructed nano-LC system, described elsewhere [20]. Briefly, 2 μl sample injections were made with an HTC-PAL autosampler (CTC Analytics AG, Zwingen, Switzerland) connected to an Agilent 1200 binary pump (Agilent Technologies, CA, USA). The peptides were trapped on a pre-column (45 × 0.075 mm i.d.) and separated on a reversed phase column, 200 × 0.050 mm. Both columns are packed in-house with 3 μm Reprosil-Pur C18-AQ particles. The flow through the analytical column was reduced by a split to approximately 100 nl/min and the gradient was as followed; 0-5 min 0.1% formic acid, 6-103 min 7-40% ACN, 0.1% formic acid, 104-108 min 40-80% ACN 0.1% formic acid.

LTQ-Orbitrap settings were: spray voltage 1.4 kV, 1 microscan for MS1 scans at 60 000 resolution (m/z 400), full MS mass range m/z 400-2000. The LTQ-Orbitrap was operated in a data-dependent mode, that is, one MS1 scan precursor ions followed by CID (collision induced dissociation) and HCD (high energy collision dissociation) MS2 scans of the three most abundant doubly or triply protonated ions in each MS1 scan. The settings for the MS2 were as follows; 1 microscan for HCD-MS2 at 7500 resolution (at m/z 400), mass range m/z 100-2000 with a collision energy of 50%, 1 microscan for CID-MS2 with a collision energy of 30%.

### Database Search and iTRAQ Quantification

MS raw data files from all 15 SCX fractions for one four-plex iTRAQ set were merged for relative quantification and identification using Proteome Discoverer version 1.2 (Thermo Fisher Scientific). The database search for each set was performed by the Mascot search engine using the following criteria: *Homo sapiens *in Swissprot version 2010_12, MS peptide tolerance as 5 ppm, MS/MS tolerance as 0.5 Da, trypsin digestion allowing two missed cleavages with variable modifications; methionine oxidation, cysteine methylthiolation, tyrosine iTRAQ4plex (+144 Da) and fixed modifications; N-terminal iTRAQ4plex, lysine iTRAQ4plex. The detected protein threshold in the software was set to 99% confidence and one peptide. The identified proteins were grouped by sharing the same sequences to minimise redundancy.

For iTRAQ quantification, the ratios of iTRAQ reporter ion intensities in MS/MS spectra (m/z 114.11-117.11) from raw data sets were used to calculate fold changes between samples. Ratios were derived by Proteome Discoverer version 1.2 using the following criteria: fragment ion tolerance as 50 ppm for the most confident centroid peak, iTRAQ reagent purity corrections factors was used, ion intensity threshold of 1000 and missing values were replaced with the minimum intensity. Only peptides unique for a given protein were considered for relative quantitation, excluding those common to other isoforms or proteins of the same family. Proteome Discoverer normalised the channel ratios and these normalised ratios were then exported into Excel for manual data interpretation.

### Bioinformatics Analysis of Proteomic Data

Identified proteins were further analysed using IPA (version 8.8) (Ingenuity^® ^Systems, http://www.ingenuity.com) to statistically determine the functions and pathways most strongly associated with the protein list. Prior to upload and analysis using IPA, the mean ratio of each quantified protein in a group was calculated and the fold change between the groups calculated. IPA utilises the Ingenuity Pathways Analysis Knowledge Base (IPA KB), a manually curated database of protein interactions from the literature, to analyse data. A dataset containing gene (or chemical) identifiers and corresponding expression values was uploaded into the application. Each identifier was mapped to its corresponding object in the IPA KB. A fold change cut-off of 1.5 was set to identify molecules whose expression was significantly differentially regulated. These proteins and their association with the IPA KB were used to generate networks and perform functional and canonical pathway analyses. The significance of the associations was assessed with the Fisher's exact test and the Benjamini-Hochberg multiple testing correction.

The PANTHER (Protein ANalysis THrough Evolutionary Relationships) Classification System [21] was used to further examine the significantly expressed molecules [22].

### Statistical Analysis

Differences and changes in clinical measurements were determined using Student's t-tests. Unpaired analyses were used for comparing naive asthmatics to controls, while paired t-tests were used for comparing pre and post treatment results. The changes due to treatment (delta change) between the two patient groups were compared using an unpaired t-test. Data are expressed as mean ± standard error of the mean (SEM) or as the mean followed by range. A p value of < 0.05 was considered statistically significant.

Student's t-tests were also used to determine significantly modified protein expression between groups. An unpaired t-test was performed on those proteins that had values for at least three samples of both healthy controls and untreated asthma. A paired t-test was performed on those proteins that had values for at least three samples of both pre and post treated groups. A p value of < 0.05 was considered statistically significant.

### Principal Component Analysis

A principal component analysis (PCA) was performed using the MultiExperiment Viewer (version 4.7), which is part of the TM4 Microarray Software Suite [23,24]. Using all proteins quantified in the dataset, the PCA was performed to visualise the relationship between the samples.

## Results

### 1. Clinical Characteristics of the Study Population

**Figure 2:**
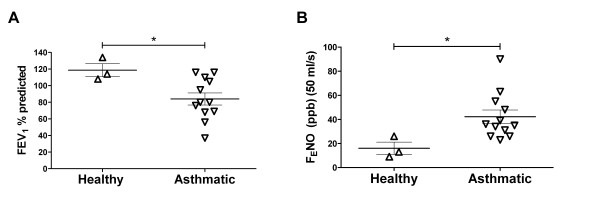
**F_E_NO and FEV_1_% predicted of healthy controls and asthmatics**. The scatter plots show the airway inflammation and lung function of healthy controls (*n *= 3) and untreated asthmatics (*n *= 12) as assessed by FEV_1_% predicted (A) and fractional exhaled nitric oxide (F_E_NO) (B). Asthmatics had significantly increased airway inflammation and significantly decreased lung function, compared to healthy controls. The bars indicate the mean ± SEM. * = indicates p value < 0.05.

The age and body mass index (BMI) of the study participants did not differ significantly between three healthy controls and the twelve untreated asthmatic patients (Table 1). Significantly reduced lung function was observed in asthmatics compared to controls in FEV_1_% predicted (p = 0.040) (Figure 2A) and FVC% predicted (p = 0.035) (Table 1). A significantly higher F_E_NO was observed in asthmatics compared to healthy controls (p = 0.04) (Figure 2B). The significant differences in FEV_1_% predicted and F_E_NO between asthmatics and controls confirmed the correct diagnosis of the individuals in this study.

**Table 1:** Clinical characteristics of healthy subjects and asthmatics

Variables	Healthy subjects	Asthmatics	p value
**Subjects **(male/female)	3 (0/3)	12 (2/10)	

**Age **(years) ^§^	55 (42-67)	57 (38-67)	0.825

**Disease duration **(years) ^§^	0	8 (0.5-20)	na

**Body Mass Index **^§^	30.9 (24.1-34.9)	31.5 (19.4-41.4)	0.897

**FVC (% pred.) **^#^	118 ± 8	86 ± 6	**0.035**

**FEV_1_/FVC **^#^	0.85 ± 0.01	0.81 ± 0.03	0.61

**PD20 **(mg of methacholine) ^#^	neg. or not performed	0.13 ± 0.05^□^	na

**Documented SPT positivity**	0	8^¤^	

The baseline clinical characteristics of all subjects showed a statistically significant difference in FVC % predicted between the two groups, using a Student's t-test. No differences were seen in the other variables. A p value of < 0.05 was considered statistically significant (shown in bold).

*Abbreviations: FEV1 = forced expiratory volume in 1 sec; FVC = forced vital capacity; PD20 = provocative dose of methacholine producing a 20% decrease in FEV1; F_E_NO = fractional exhaled nitric oxide; SPT = skin prick test; na = not applicable; ^§ ^= data presented as mean (range); ^# ^= data presented as mean ± standard error of the mean; *^□ ^= *n = 10 (1 patient was negative, 1 patient was not tested) ^¤ ^= 2 patients were negative, 2 patients were not tested, but had no clinical history of allergy*.

### 2. Clinical Effects of Treatment with Budesonide

In those asthmatics patients who underwent treatment with placebo or budesonide, and who consented to both pre and post biopsies, the age, disease duration and BMI were not significantly different between the two groups (Table 2). The four asthmatic patients treated with placebo showed no significant changes in lung function parameters, F_E_NO or methacholine reactivity, between their pre and post treatment visits (Table 2). Treatment with budesonide in three patients, resulted in a significant increase in FEV_1_% predicted (p = 0.017), with other parameters showing no significant changes between their pre and post treatment visits (Table 2). The change in clinical measurements between the pre and post treatment visits (delta change) of placebo and budesonide treated patients was not significantly different (Table 2).

**Table 2:** Clinical baseline and treatment effects of asthmatics receiving placebo or budesonide

	Placebo			Budesonide		p value	
**Subjects **(male/female)	4 (1/3)			3 (0/3)			

**Age **(years) ^§^	52 (38-64)			65 (62-67)		0.091	

**Disease duration **(years) ^§^	9 (0.5-20)			9 (5-17)		0.951	

**Body Mass Index **^§^	29.4 (21.8-34.7)			32.0 (28.9-37.0)		0.578	

**Documented SPT positivity**	3*^¤^*			2*^¤¤^*			

	**Pre**	**Post**	**p value**	**Pre**	**Post**	**p value**	Δ **p value**

**FEV_1 _(% pred.) **^#^	66 ± 12.52	75 ± 8.67	0.662	88 ± 14.42	101 ± 15.72	**0.017**	0.838

**FVC (% pred.) **^#^	78 ± 13.46	84 ± 8.95	0.738	87 ± 13.78	101 ± 15.01	0.143	0.735

**FEV_1_/FVC **^#^	0.71 ± 0.06	0.76 ± 0.07	0.472	0.85 ± 0.09	0.83 ± 0.05	0.676	0.424

**PD20 **(mg of methacholine) ^#^	0.077 ± 0.06*	0.084 ± 0.06	0.382	0.243 ± 0.14	-^†^	na	na

**F_E_NO **^#^	53 ± 14.85	40 ± 9.76	0.087	46 ± 5.84	32 ± 9.14	0.092	0.880

The demographics of the asthmatics receiving placebo treatment (*n *= 4) and budesonide treatment (*n *= 3) showed no significant differences in age, disease duration or body mass index, as determined by a Student's t-test. The clinical measurements, pre and post treatment, were compared using a paired t-test and showed no changes as a result of placebo treatment. Budesonide treatment resulted in a statistically increased FEV_1_% predicted. No significant differences were seen for the other variables measured. A p value of < 0.05 was considered statistically significant (shown in bold).

Abbreviations: FEV_1 _= forced expiratory volume in 1 sec; FVC = forced vital capacity; PD20 = provocative dose of methacholine producing a 20% decrease in FEV_1_; F_E_NO = fractional exhaled nitric oxide; SPT = skin prick test; ^§ ^= data presented as mean (range); ^¤ ^= 1 patient was SPT negative; ^¤¤ ^= SPT was not performed on 1 patient, but they had no clinical history of allergy; ^# ^= data presented as mean ± standard error of the mean; na = not applicable;* = PD20 measurements were only available for three patients; † = PD20 measurement was only available for one patient; Δ = delta change.

### 3. Differences in the Bronchial Proteome of Asthmatics Compared to Healthy Subjects

#### 3.1 Protein Identification

A union of the three healthy controls and twelve untreated asthmatic samples identified and quantified 1734 unique proteins relative to the reference pool (99% confidence, one peptide, false discovery rate at p < 0.01; 1.14-7.04%). Of these, 3% were identified in all 15 samples. Of the 1734 proteins, 603 proteins were common to both asthmatics and healthy, while 65 proteins were unique to healthy subjects and 1066 were unique to asthmatics.

#### 3.2 Significantly Modified Proteins

To determine differentially expressed proteins between healthy controls and untreated asthmatics, t-tests were performed on valid proteins. Of the 169 proteins that were possible to analyse statistically, seven were identified as significantly different (Table 3).

**Table 3:** The significantly differentially expressed proteins in untreated asthmatics compared to healthy controls

**Swiss Prot Acc. No**.	Symbol	Entrez Gene Name	Fold Change	Location	Protein Class
P08758	ANXA5	annexin A5	-1.98	Plasma membrane	transfer/carrier protein

Q07507	DPT	dermatopontin	-2.15	Extracellular space	extracellular matrix binding protein

Q96KK5	HIST1H2AH	histone cluster 1, H2ah	-2.85	Nucleus	histone

P02545	LMNA	lamin A/C	-1.85	Nucleus	structural protein

P62937	PPIA	peptidylprolyl isomerase A (cyclophilin A)	-1.64	Cytoplasm	isomerase

P18124	RPL7	ribosomal protein L7	-1.82	Cytoplasm	ribosomal protein

P62917	RPL8	ribosomal protein L8	-1.76	Cytoplasm	ribosomal protein

^© ^2000-2011 Ingenuity Systems, Inc. All rights reserved.

All listed proteins showed a statistically significant difference (p value < 0.05) between the groups using a Student's t-test. The fold change for each protein is calculated by dividing the average ratio of the untreated asthmatics with the average ratio of the healthy controls. The negative fold change indicates down regulation of protein expression in asthmatic patients. The cell location of the protein is annotated by IPA. The protein class is determined using the PANTHER Classification System.

These seven proteins were mapped in IPA and displayed a range of protein classes and functions, including cellular movement and immune cell trafficking. Specifically, the IPA KB detailed roles including collagen fibrillogenesis (dermatopontin (DPT; Q07507)), protein elongation (ribosomal protein L7, -L8, (RPL7; P18124, RPL8; P62917)) and chemotaxis (peptidylprolyl isomerase A (cyclophilin A)(PPIA; P62937)). Three of the molecules have been previously described in relation to asthma, fibrosis and inflammation, including annexin 5A (ANXA5; P08758) [25], lamin A/C (LMNA;P02545) [26,27] and PPIA [28], indicating the sensitivity of the method and clinical relevance of the samples.

#### 3.3 Analysis of Functions, Pathways and Networks in Asthmatics

**Figure 3:**
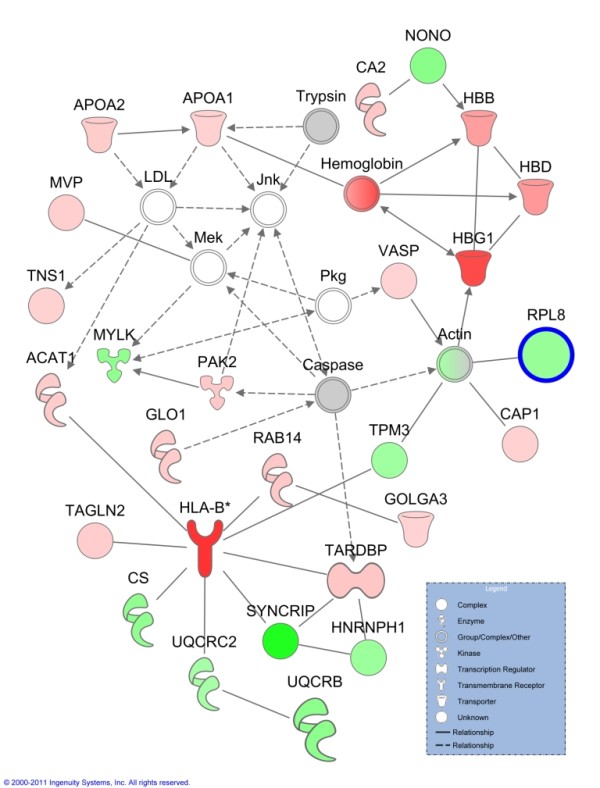
**Network of differentially expressed proteins in untreated asthmatics compared to healthy controls**. The top network generated by Ingenuity Pathways Analysis from quantified proteins (1.5 fold change threshold) of untreated asthmatics compared to healthy controls, contains the functions of haematological system development and function, lipid metabolism and molecular transport. Molecules with bold outline are significantly different between the two groups using a Student's t-test. Lines indicate relationships between molecules. Arrows at the end of these lines indicated the direction of the interaction. Molecules that are up-regulated and down-regulated in the dataset are coloured red and green respectively. Grey molecules do not meet the cut-off threshold. Uncoloured molecules have been added from the Ingenuity Knowledge Base. *Entrez gene names for molecules are: ACAT1 = acetyl-CoA acetyltransferase 1, APOA1 = apolipoprotein A-I, APOA2 = apolipoprotein A-II, CA2 = carbonic anhydrase II, CAP1 = CAP, adenylate cyclase-associated protein 1 (yeast), CS = citrate synthase, GLO1 = glyoxalase I, GOLGA3 = golgin A3, HBB = hemoglobin (beta), HBD = hemoglobin (delta), HBG1 = hemoglobin (gamma A), HLA-B = major histocompatibility complex, class I, B, HNRNPH1 = heterogeneous nuclear ribonucleoprotein H1 (H), MVP = major vault protein, MYLK = myosin light chain kinase, NONO = non-POU domain containing, octamer-binding, PAK2 = p21 protein (Cdc42/Rac)-activated kinase 2, RAB14 = RAB14, member RAS oncogene family, RPL8 = ribosomal protein L8, SYNCRIP = synaptotagmin binding, cytoplasmic RNA interacting protein, TAGLN2 = transgelin 2, TARDBP = TAR DNA binding protein, TNS1 = tensin 1, TPM3 = tropomyosin 3, UQCRB = ubiquinol-cytochrome c reductase binding protein, UQCRC2 = ubiquinol-cytochrome c reductase core protein II, VASP = vasodilator-stimulated phosphoprotein*.

To examine the protein functions and pathways of the entire proteome, the protein ratio of asthmatics compared to healthy subjects, was analysed using IPA. The highest scoring network of proteins included the functions of "haematological system development and function", "lipid metabolism" and "molecular transport". This network included haemoglobins, apolipoproteins 1 and MHC class I (Figure 3). The network also included RPL8, a protein involved in protein synthesis, which was identified as significantly different between healthy controls and asthmatics. In addition, IPA analysis of the differentially expressed proteins of untreated asthmatics identified associations with the biological functions of "respiratory disease", "cell to cell signalling", "haematological system development and function" and "tissue development" (data not shown). The top canonical pathways associated with proteins from asthmatics included "acute phase response signalling" and "intrinsic prothrombin activation pathway", while the toxicology lists include "positive acute phase response proteins" "oxidative stress" and "negative acute phase response proteins" (data not shown). The association between the molecules differentially expressed in the asthmatics and the functions, networks and pathways above suggest increased haematological involvement and increased oxidative stress in asthmatics compared to controls.

### 4. The Effect of Placebo vs Budesonide on the Bronchial Proteome of Asthmatics

#### 4.1 Protein Identification

In the biopsies from placebo treated asthmatics, 1333 proteins were quantified in at least one of the four pre placebo biopsies, with 9.2% being quantified in all four. In the post placebo biopsy proteomes, 683 proteins were quantified, with 9.8% being quantified in all four samples. Of the 1446 unique proteins identified in at least one of these samples, 570 were common to both the pre and post placebo proteome, while 763 proteins were unique to the pre placebo samples and 113 proteins unique to the post placebo samples. Examining the paired biopsies, 58 proteins were quantified in both pre and post placebo biopsies of all four asthmatics.

In biopsies from budesonide treated asthmatics, 1234 proteins were quantified in at least one of the three pre budesonide biopsies, with 14.7% of proteins quantified in all three biopsy proteomes. In post budesonide samples, a total of 854 proteins were quantified in at least one of three post budesonide biopsies, with 29.9% of proteins quantified in all three individuals. Of the 1419 unique proteins identified in at least one of these samples, 669 proteins were common to both the pre and post budesonide proteome, while 565 proteins were unique to the pre budesonide samples and 185 proteins were unique to the post budesonide samples. Comparing the paired pre and post budesonide biopsies, 141 proteins were quantified in both biopsies of all three subjects.

In all placebo and budesonide treated individuals, 54 proteins were quantified in all asthmatics, in both their pre and post treatment biopsies.

#### 4.2 Significantly Modified Proteins

Of the 115 proteins that could be statistically analysed for placebo treated patients, three were identified as significantly different between pre and post placebo biopsies (Table 4), while seven of 141 proteins were identified as significantly modified between pre and post budesonide biopsies (Table 5). The proteins that changed significantly during placebo treatment were different from those significantly modified with budesonide treatment.

**Table 4:** Significantly modified proteins in post treated placebo versus pre treated placebo asthmatic patients

**Swiss Prot Acc. No**.	Symbol	Entrez Gene Name	Fold Change	Location	Protein Class
P60709	ACTB	actin, beta	-1.66	Cytoplasm	actin and actin related protein

P05783	KRT18	keratin 18	1.31	Cytoplasm	structural protein

P01009	SERPINA1	serpin peptidase inhibitor, clade A (alpha-1 antiproteinase, antitrypsin), member 1	2.34	Extracellular space	serine protease inhibitor

^© ^2000-2011 Ingenuity Systems, Inc. All rights reserved.

All listed proteins showed a statistically significant difference (p value < 0.05) between the groups using a Student's t-test. The fold change for each protein is calculated by dividing the average ratio of the placebo post treated biopsy with the average ratio of the paired pre placebo biopsy. The negative fold change indicates down regulation of protein expression by placebo, while the positive fold change indicates up regulation by placebo. The cell location of the protein is annotated by IPA. The protein class is determined using the PANTHER Classification System.

**Table 5:** Significantly modified proteins in post treated budesonide versus pre treated budesonide asthmatics

**Swiss Prot Acc. No**.	Symbol	Entrez Gene Name	Fold Change	Location	Protein Class
P01023	A2M	alpha-2-macroglobulin	-1.27	Extracellular space	cytokine

P04075	ALDOA	aldolase A, fructose-bisphosphate	-1.22	Cytoplasm	aldolase

P06576	ATP5B	ATP synthase, H+ transporting, mitochondrial F1 complex, beta polypeptide	-1.76	Cytoplasm	ATP synthase

Q9BPU6	DPYSL5	dihydropyrimidinase-like 5	2.01	Cytoplasm	hydrolase

P60866	RPS20	ribosomal protein S20	1.54	Cytoplasm	ribosomal protein

P29508	SERPINB3	serpin peptidase inhibitor, clade B (ovalbumin), member 3	2.31	Extracellular space	serine protease inhibitor

P08670	VIM	vimentin	-1.69	Cytoplasm	structural protein

^© ^2000-2011 Ingenuity Systems, Inc. All rights reserved.

All listed proteins showed a statistically significant difference (p value < 0.05) between the groups using a Student's t-test. The fold change for each protein is calculated by dividing the average ratio of the budesonide post treated biopsy with the average ratio of the paired pre budesonide biopsy. The negative fold change indicates down regulation of protein expression after budesonide treatment, while the positive fold change indicates up regulation after budesonide treatment. The cell location of the protein is annotated by IPA. The protein class is determined using the PANTHER Classification System.

The significantly changed proteins in the placebo treated group reflect the natural progression of disease (Table 4). The proteins have a variety of functions, annotated by the IPA KB, including cell movement (actin, beta (ACTB; P60709), the disruption of microtubules (keratin 18 (KRT18; P05783)) and production of reactive oxygen species (serpin peptidase inhibitor, clade A, member 1 (SERPINA1; P01009)). The increased expression of SERPINA1 suggests an increase in inflammatory processes in placebo treated patients, while the decreased expression of ACTB suggests dysregulated growth and proliferation.

The significantly modified proteins in the budesonide treated proteome may reflect the proteomic changes induced by the glucocorticoid (Table 5). Of the seven proteins, several were involved in inflammatory and immunological disorders; alpha-2-macroglobulin (A2M; P01023), aldolase A (ALDOA; P04075), serpin peptidase inhibitor, clade B, member 3 (SERPINB3; P29508), vimentin (VIM; P08670)), as well as cell movement (SERPINB3) and energy production (ATP synthase, H+ transporting, mitochondrial F1 complex, beta polypeptide (ATP5B; P06576). Of interest, a cytokine, A2M, was identified and quantified. The quantification of several cytokines identified in this study, including secretoglobin, family 1A, member 1 (SCGB1A1; P11684) and tumour necrosis factor (ligand) superfamily, member 13b (TNFSF13B; Q9Y275), indicates the sensitivity of the proteomics method used. The decreased expression of A2M and VIM suggests possible suppression of inflammation in response to budesonide.

#### 4.3 Analysis of the Effect of Treatment on Functions and Pathways

**Figure 4:**
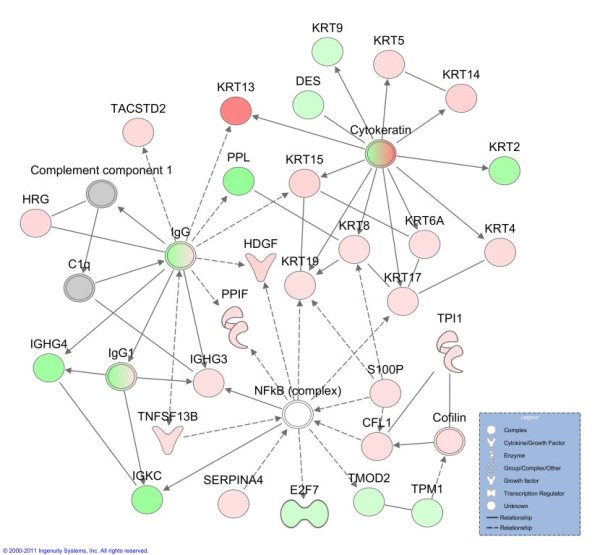
**Network of differentially expressed proteins of post placebo compared to paired pre placebo biopsy**. The top network generated by Ingenuity Pathways Analysis from quantified proteins of post placebo asthmatics compared to their pre placebo biopsy. The network contains the functions of hair and skin development and function, organ development, dermatological diseases and conditions. Molecules with bold outline are significantly different between the two groups using a Student's t-test. Lines indicate relationships between molecules. Arrows at the end of these lines indicated the direction of the interaction. Molecules that are up-regulated and down-regulated in the dataset are coloured red and green respectively. Grey molecules do not meet the cut-off threshold. Uncoloured molecules have been added from the IPA KB. *Entrez gene names for molecules are: CFL1 = cofilin 1 (non-muscle) DES = desmin, E2F7 = E2F transcription factor 7, HDGF = hepatoma-derived growth factor, HRG = histidine-rich glycoprotein, IGHG3 = immunoglobulin heavy constant gamma 3 (G3 m marker), IGHG4 = immunoglobulin heavy constant gamma 4 (G4 m marker), IGKC = immunoglobulin kappa constant, KRT2 = keratin 2, KRT4 = keratin 4, KRT5 = keratin 5, KRT8 = keratin 8, KRT9 = keratin 9, KRT13 = keratin 13, KRT14 = keratin 14, KRT15 = keratin 15, KRT17 = keratin 17, KRT19 = keratin 19, KRT6A = keratin 6A, PPIF = peptidylprolyl isomerase F, PPL = periplakin, S100P = S100 calcium binding protein P, SERPINA4 = serpin peptidase inhibitor, clade A (alpha-1 antiproteinase, antitrypsin), member 4, TACSTD2 = tumor-associated calcium signal transducer 2, TMOD2 = tropomodulin 2 (neuronal), TNFSF13B = tumor necrosis factor (ligand) superfamily, member 13b, TPI1 = triosephosphate isomerase 1, TPM1 = tropomyosin 1 (alpha)*.

The quantified proteins of the paired biopsies treated with either placebo or budesonide were analysed using IPA. The analysis of the post placebo proteins changed more than 1.5 fold compared to the pre placebo control biopsy, revealed that the top network contained the functions "hair and skin development and function", "organ development" and "dermatological diseases and conditions" (Figure 4). The network displays three distinct nodes; the NF-kB complex, the IgG complex and the cytokeratin complex.

The most relevant biological functions following placebo treatment included "gastrointestinal disease", "cell-to-cell signalling and interaction" and "tissue development", while top canonical pathways showed an association with actin-based signalling pathways, suggesting increased structural modification events. This was then followed by the acute phase response and prothrombin activation pathways.

**Figure 5:**
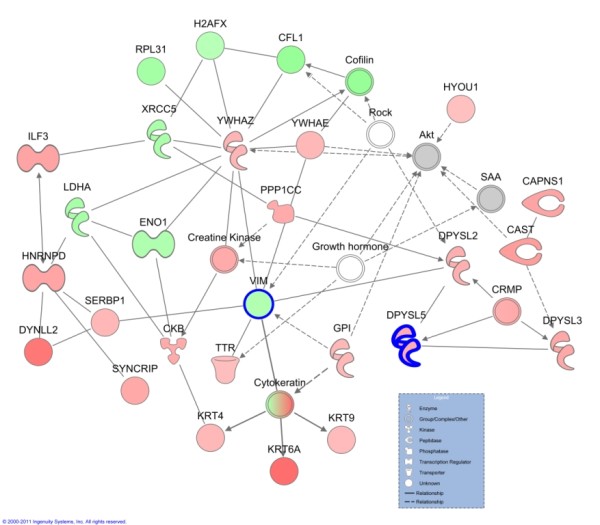
**Network of differentially expressed proteins of post budesonide compared to paired pre treatment biopsy**. The top network generated by Ingenuity Pathways Analysis from quantified proteins of post budesonide asthmatics compared to their pre placebo biopsy. The network contains the functions of cancer, genetic disorder and respiratory disease. Molecules with bold outline are significantly different between the two groups using a Student's t-test. Lines indicate relationships between molecules. Arrows at the end of these lines indicated the direction of the interaction. Molecules that are up-regulated and down-regulated in the dataset are coloured red and green respectively. Grey molecules do not meet the cut-off threshold. Uncoloured molecules have been added from the IPA KB. *Entrez gene names for molecules are: CAPNS1 = calpain, small subunit 1, CAST = calpastatin, CFL1 = cofilin 1 (non-muscle), CKB = creatine kinase, brain, DPYSL2 = dihydropyrimidinase-like 2, DPYSL3 = dihydropyrimidinase-like 3, DPYSL5 = dihydropyrimidinase-like 5, DYNLL2 = dynein, light chain, LC8-type 2, ENO1 = enolase 1, (alpha), GPI = glucose-6-phosphate isomerase, H2AFX = H2A histone family, member X, HNRNPD = heterogeneous nuclear ribonucleoprotein D (AU-rich element RNA binding protein 1, 37 kDa), HYOU1 = hypoxia up-regulated 1, ILF3 = interleukin enhancer binding factor 3, 90 kDa, KRT4 = keratin 4, KRT9 = keratin 9, KRT6A = keratin 6A, LDHA = lactate dehydrogenase A, PPP1CC = protein phosphatase 1, catalytic subunit, gamma isozyme, RPL31 = ribosomal protein L31, SERBP1 = SERPINE1 mRNA binding protein 1, SYNCRIP = synaptotagmin binding, cytoplasmic RNA interacting protein, TTR = transthyretin, VIM = vimentin, XRCC5 = X-ray repair complementing defective repair in Chinese hamster cells 5 (double-strand-break rejoining), YWHAE = tyrosine 3-monooxygenase/tryptophan 5-monooxygenase activation protein, epsilon polypeptide, YWHAZ = tyrosine 3-monooxygenase/tryptophan 5-monooxygenase activation protein, zeta polypeptide*.

Budesonide is a common anti-inflammatory glucocorticoid used to treat asthma. "Cell movement", "cell growth and proliferation", and "cell to cell signalling and interaction" were the top biological functions associated with post budesonide asthma, compared to pre budesonide. The top network of post budesonide proteins included the functions "cancer", "genetic disorder" and "respiratory disease" (Figure 5). This network includes two proteins which were determined to be significantly modified in response to treatment. VIM is a cytoskeletal protein, while dihydropyrimidinase-like 5 (DPYSL5; Q9BPU6) plays a role in growth and guidance.

The most relevant canonical pathways following budesonide treatment included prothrombin activation pathways followed by actin motility and signalling, suggesting an involvement in vascular exudation and remodelling. This is further confirmed by the top toxicology list association of hepatic fibrosis (data not shown). Specifically, the decreased expression of some collagen proteins suggests that the remodelling process is being suppressed.

Comparing the placebo and budesonide analyses, placebo treatment was generally associated with tissue development and actin based signalling, which was different to the budesonide treated with more associations to cellular functions and prothrombin activation (data not shown). Importantly, the placebo treatment was associated with hepatic fibrosis and positive acute phase proteins. These associations were reduced in budesonide treatment, with a drastically decreased association with acute phase proteins.

A PCA was performed in order to visualise the relationship between the 22 samples, including healthy controls, untreated asthmatics, placebo treated asthmatics and budesonide treated asthmatics. A total of 1833 unique proteins were identified in at least one of the 22 samples in the dataset and the expression data analysed by PCA. Principal components 1 and 2 represented 37.47% of the overall variability of the data. A biplot of these two components revealed a tight cluster of healthy controls, within a larger cluster of asthmatics. The proteome of two untreated asthmatics, two post placebo asthmatics and one post budesonide asthmatic separate from the main cluster (data not shown). The PCA suggests minimal variation in the proteome of the healthy controls and demonstrated the known heterogeneity of asthma.

## Discussion

There has been rapid growth in the identification of biomarkers and mechanisms using proteomic methods. Clinical proteomics is challenged by limited patient numbers and limited patient material. This study has shown for the first time that bronchial biopsy material can contain sufficient material for quantitative proteomic analysis. In addition, the individual proteomes of patients can be determined and features of asthma, including structural changes and cellular movement, can be identified.

The current study has shown for the first time that small clinically relevant tissue samples (bronchial biopsies) can be used to successfully analyse the proteome of patients with asthma. By combining iTRAQ^® ^technology with sensitive LC-MS/MS, proteins extracted from endobronchial biopsies were identified and it was possible to quantify characteristics of asthma, including the expression of proteins involved in oxidative stress, immune cell trafficking and inflammatory responses. Importantly, we also show that treatment with an inhaled glucocorticoid significantly affects the proteome and associated relevant functions, again arguing that quantitative proteomic studies can identify disease relevant processes in the bronchi and can reflect responses to treatment.

The number of patients utilised for this proteomics study was relatively low, as bronchial biopsies were not available from all subjects due to some declining to undergo bronchoscopies. As this was not anticipated, it is unlikely that a selection bias for the bronchial biopsy results was introduced. Despite the low numbers, a significant difference in several measurements was found between the studied groups and the paired samples add strength to the study. It is interesting to observe that both FEV_1% _predicted and F_E_NO were significantly different in the asthma group compared with the controls, confirming the correct diagnosis of the individuals. Despite being far from powered, the study was able to determine clinical effects of inhaled glucocorticoids, as observed by an improvement in percent predicted FEV_1 _by the applied treatment, again confirming the correct diagnosis and clinically relevant treatment effects. Future screening studies of this depth will probably be conducted in small patient subsets, due to the invasive nature of the sampling. The current study is not only the first to utilise quantitative proteomics in bronchial biopsies in asthma, but also the largest study determining the proteome of asthma.

The bronchial biopsies from the asthmatics, taken through fibreoptic bronchoscopes, are a few millimetres in size, subsequently resulting in a small yield of extracted protein. Despite these limitations, the coupling of the iTRAQ^® ^technology and the nanoLC-LTQ-Orbitrap XL instrument successfully identified a large number of proteins in the samples, including many that were relevant to asthma and respiratory diseases. As might be expected from tissue samples, the currently applied technique was able to identify and quantify a larger number of proteins than any previous proteomic study in asthma using BALF [10] or specific cells [11,29], despite no affinity depletion of high abundance proteins. Interestingly, the low abundance of cytokines usually prevents their quantification in proteomics studies. However, this study has identified and quantified several cytokines in bronchial biopsies, including SCGB1A1 and TNFSF13B. These two cytokines had increased expression with placebo treatment, which was reduced in budesonide treatment. SCGB1A1 is a transient marker of inflammation [30], while TNFSF13B has been associated with both Th1 and Th2 inflammation [31,32]. Other cytokines identified, but not quantified, included interleukin 6, chemokine (C-C motif) ligand 5 (CCL5; P13501) and macrophage migration inhibitory factor (MIF; P14174), again documenting the relatively high sensitivity of the proteomics method utilised. Currently, no proteomics method will identify and quantify all proteins present in a biological sample. The identification of low abundant proteins such as cytokines and other regulatory molecules, which may have profound biological effects at low concentrations, may require an even more sensitive methodology to be adequately measured. It is possible that with exclusion lists of irrelevant proteins, many more low abundance proteins could be identified [33]. In addition, multiple reaction monitoring mass spectrometry may also allow the identification of low abundance proteins, as well as serve as a method for validation [34].

Both the significant proteins, as well as the IPA results, show clear differences in the proteome of bronchial biopsies of asthmatics vs healthy controls. Asthma relevant proteins that were shown to be different in asthmatics vs controls included SCGB1A1, SERPINA1, thrombins and fibrinogens. LMNA, one of the significantly differentially expressed proteins of asthmatics, has been shown to be decreased in fibroblasts from fibrotic lungs compared to non-fibrotic lungs [27]. The altered expression of these proteins in the bronchi of asthmatics supports the evidence of inflammatory process in the airways of the studied individuals. Interestingly, the top network expressed in asthma vs controls connects "haematological system development and function" with "lipid metabolism" and "molecular transport".

After treatment with placebo, the proteomic analysis showed some significantly modified proteins between post and pre treatment. The expression of these proteins may occur for two possible reasons. Firstly, they may be due in part to inherent experimental variation, perhaps depending on slight differences in the cellular composition in the biopsies. Secondly, the changes during placebo treatment could be due to the natural progression of disease over the treatment period.

Treatment with budesonide changes protein expression to a larger extent than placebo treatment and several of these changed proteins, including fibronectin (FN1; P02751) and SCGB1A1, are likely to be related to effects of the glucocorticoid. The top network of molecules generated for budesonide treatment included "cancer", "genetic disorder" and "respiratory disease" and includes proteins such as keratin 6A (KRT6A; P02538), dihydropyrimidinase-like 5 (DPYSL5; Q9BPU6), and interleukin enhancer binding factor 3 (ILF3; Q12906) which had increased expression, while downregulated proteins included cofilin 1(CFL1; P23528), alpha enolase 1(ENO1; P06733) and VIM (Figure 5). ILF3, has recently been described as a regulator of HS4-dependent IL13 transcription, an important cytokine in Th2 immune responses [35]. A decreased expression of VIM, a mesenchymal marker, may suggest reduced remodelling in response to budesonide, as epithelial to mesenchymal transition has been seen to contribute to remodelling [36,37]. With roles in inflammation and remodelling, these proteins will need to be validated and further investigated as potential targets.

Inhaled glucocorticoids are potent anti-inflammatories recommended for asthmatics. Budesonide has been seen to reduce inflammatory cells [38] and correct gene expression [39] in bronchial biopsies, however, global proteome changes had not been analysed. A biological function that is significantly affected by inhaled budesonide is "immune cell trafficking", in addition to the functions of "tissue development" and "haematological systems development and function", which was observed to change during placebo treatment and is also different between untreated asthma and controls. The general anti-inflammatory effect of budesonide can be seen with the improved lung function of the asthmatics and with the reduced association with acute phase proteins. Thus, our data show that protein networks and functions relevant, or likely to be relevant, to asthma, are altered by inhaled glucocorticoids.

The application of iTRAQ^® ^technology with LC-MS/MS has been used in several other diseases and samples [14,15]. This study is the first to describe the application of this method to examine the asthmatic bronchial biopsy proteome and effects of glucocorticoid treatment. Studying the proteome of asthma is a complicated task, for several reasons. For one, the heterogeneity of asthma argues the existence of several different subtypes of disease and the current study is too small to include sub-classifications of disease [40]. The principal component analysis allowed the visualisation of the relationship between the samples of this study. While the healthy controls clustered together, as anticipated, the asthmatic samples showed more variability. This may reflect the known variability of the disease, with its multiple phenotypes [41]. The budesonide treated samples did not cluster together, which is not unexpected, as the response the inhaled glucocorticoids can vary substantially between asthmatic patients [42]. Therefore, this study reflects the mechanism and features of asthma in general and will need to be expanded in phenotypes of asthma to further dissect the mechanisms of disease.

A limitation of the current study is the experimental and biological variation occurring at multiple levels. This is true for any clinical proteomics study of clinical proteomics in disease, but is especially relevant in asthma, a disease with extensive variability in intrinsic biological activity over time. It must be cautioned that to develop relevant biomarkers of disease from the current study, the results will need to be further validated by other methods in order to address the limitations of potential false discovery, small sample size and gender specific proteome differences. Following this, verification will be required in an independent group of patients, as well as other inflammatory and respiratory diseases. While the findings of the current study have not been validated, the results from the IPA support their involvement in processes associated with asthma, including structural changes, collagen accumulation and angiogenesis [43].

## Conclusions

This extensive proteomic study has identified global mechanisms and features common to asthma and has determined for the first time that these features can be identified from small biopsy samples of individuals with the disease. However, the limited patient number and heterogeneity of asthma argues that even more detailed studies will be required in the future to identify new mechanisms of disease. Thus, to further understand the complexities of asthma in the many phenotypes that are displayed, well characterised subsets of patients, representing different phenotypes of asthma will need to be examined.

## List of Abbreviations

2-DE: two-dimensional electrophoresis; ACN: acetonitrile; BALF: bronchoalveolar lavage fluid; BMI: body mass index; CHAPS: 3-[(3-cholamidopropyl)dimethylammonio]-1-propanesulphonate; CID: collision induced dissociation; EDTA: ethylenediaminetetraacetic acid; F_E_NO: fractional exhaled nitric oxide; FEV1: forced expiratory volume in 1 sec; FVC: forced vital capacity; HCD: high energy collision dissociation; IPA: Ingenuity Pathway Analysis; IPA KB: Ingenuity Pathway Analysis Knowledge Base; iTRAQ: isobaric tags for relative and absolute quantitation; LC-MS/MS: liquid chromatography tandem mass spectrometry; MALDI-TOF: matrix-assisted laser-desorption ionization time-of-flight; MMTS: methyl methanethiolsulfonate; MS: mass spectrometry; *m/z *mass-to-charge ratio; nanoLC-LTQ: nano liquid chromatography linear trap quadrupole; nano LC-MS/MS: nano liquid chromatography tandem mass spectrometry; PANTHER: Protein ANalysis THrough Evolutionary Relationships; PD20: provocative dose of methacholine producing a 20% decrease in FEV1; SCX: strong cation exchange; SDS-PAGE: sodium dodecyl sulphate polyacrylamide gel electrophoresis; SELDI-TOF: surface-enhanced laser desorption/ionization time-of-flight; SEM: standard error of the mean; SPT: skin prick test; TEAB: triethylammonium bicarbonate; TCEP: ((tris(2-carboxyethyl)phosphine).

## Competing interests

The authors declare that they have no competing interests.

## Authors' contributions

BS, AB, AK, KSB and RS conducted the randomised trial, including biopsy sampling. LE participated in the design of the proteomic study. EC and CS participated in the proteomic experimental design and performed the proteomic experiments. SO participated in the design of the proteomic study, analysed the patient and proteomic data and drafted the manuscript. JL conceived the proteomics study, participated in the design and helped draft the manuscript. All authors approved the final manuscript.
